# S-nitrosation of mitochondrial connexin 43 regulates mitochondrial function

**DOI:** 10.1007/s00395-014-0433-x

**Published:** 2014-08-13

**Authors:** Daniel Soetkamp, Tiffany T. Nguyen, Sara Menazza, Christine Hirschhäuser, Ulrike B. Hendgen-Cotta, Tienush Rassaf, Klaus D. Schlüter, Kerstin Boengler, Elizabeth Murphy, Rainer Schulz

**Affiliations:** 1 Systems Biology Center, National Heart, Lung, and Blood Institute, National Institutes of Health, Bethesda, MD USA; 2 Physiologisches Institut, Fachbereich Medizin der Justus-Liebig-Universität Giessen, Aulweg 129, 35392 Giessen, Germany; 3 Department of Medicine, Division of Cardiology, Pulmonology and Vascular Medicine, Heinrich-Heine-University Düsseldorf, Moorenstr 5, 40225 Düsseldorf, Germany

**Keywords:** Cardioprotection, Connexin hemichannel, Ischemic preconditioning (IPC), S-nitrosation (SNO), Nitric oxide (NO)

## Abstract

S-nitrosation (SNO) of connexin 43 (Cx43)-formed channels modifies dye uptake in astrocytes and gap junctional communication in endothelial cells. Apart from forming channels at the plasma membrane of several cell types, Cx43 is also located at the inner membrane of myocardial subsarcolemmal mitochondria (SSM), but not in interfibrillar mitochondria (IFM). The absence or pharmacological blockade of mitochondrial Cx43 (mtCx43) reduces dye and potassium uptake. Lack of mtCx43 is associated with loss of endogenous cardioprotection by ischemic preconditioning (IPC), which is mediated by formation of reactive oxygen species (ROS). Whether or not mitochondrial Lucifer Yellow (LY), ion uptake, or ROS generation are affected by SNO of mtCx43 and whether or not cardioprotective interventions affect SNO of mtCx43 remains unknown. In SSM from rat hearts, application of NO donors (48 nmol to 1 mmol) increased LY uptake (0.5 mmol SNAP 38.4 ± 7.1 %, *p* < 0.05; 1 mmol GSNO 28.1 ± 7.4 %, *p* < 0.05) and the refilling rate of potassium (SNAP 227.9 ± 30.1 %, *p* < 0.05; GSNO 122.6 ± 28.1 %, *p* < 0.05). These effects were absent following blockade of Cx43 hemichannels by carbenoxolone as well as in IFM lacking Cx43. Unlike potassium, the sodium permeability was not affected by application of NO. Furthermore, mitochondrial ROS formation was increased following NO application compared to control SSM (0.5 mmol SNAP 22.9 ± 1.8 %, *p* < 0.05; 1 mmol GSNO 40.6 ± 7.1 %, *p* < 0.05), but decreased in NO treated IFM compared to control (0.5 mmol SNAP 14.4 ± 4 %, *p* < 0.05; 1 mmol GSNO 13.8 ± 4 %, *p* < 0.05). NO donor administration to isolated SSM increased SNO of mtCx43 by 109.2 ± 15.8 %. Nitrite application (48 nmol) to mice was also associated with elevated SNO of mtCx43 by 59.3 ± 18.2 % (*p* < 0.05). IPC by four cycles of 5 min of ischemia and 5 min of reperfusion increased SNO of mtCx43 by 41.6 ± 1.7 % (*p* < 0.05) when compared to control perfused rat hearts. These data suggest that SNO of mtCx43 increases mitochondrial permeability, especially for potassium and leads to increased ROS formation. The increased amount of SNO mtCx43 by IPC or nitrite administration may link NO and Cx43 in the signal transduction cascade of cardioprotective interventions.

**Electronic supplementary material:**

The online version of this article (doi:10.1007/s00395-014-0433-x) contains supplementary material, which is available to authorized users.

## Introduction

Connexin 43 (Cx43) is localized at the plasma membrane of several cell types, including astrocytes, endothelial cells, and the sarcolemma of cardiomyocytes. Six Cx43 proteins assemble to form hemichannels, which contribute to cellular volume regulation and release of signaling molecules into the extracellular fluid. Two opposing hemichannels of neighboring cells can form a pore and many pores form a gap junction (GJ) [55]. Application of nitric oxide (NO) at low concentrations increases Cx43 hemichannel permeability to hydrophilic fluorescent molecules in astrocytes, an effect that is abolished by hemichannel blockers [49]. In addition, intercellular communication in the vessel wall is regulated by S-nitrosation (SNO); SNO is a reversible redox-dependent protein modification in which a NO group is covalently attached to the free thiol group of cysteine residues [15]. Indeed, SNO of the cysteine residue 271 increases and denitrosation decreases permeability of Cx43 myoendothelial gap junctions [66].

Besides its location at the sarcolemma, Cx43 is also present at the inner membrane of cardiomyocyte subsarcolemmal mitochondria (SSM), but not in interfibrillar mitochondria (IFM) [1, 5, 54]. In vitro cross-linking studies on mitochondria show complexes of a molecular weight comparable with that of Cx43 hemichannels [36]. Supporting this notion, the two hemichannel blockers carbenoxolone and heptanol reduce mitochondrial uptake of Lucifer Yellow (LY) dye [36]. Mitochondrial Cx43 (mtCx43) influences mitochondrial respiration, potassium (K^+^) influx [3, 4, 6] and reactive oxygen species (ROS) production. Lack of mtCx43 abolishes myocardial infarct size reduction by ischemic preconditioning; thus mtCx43 appears to be an essential part of the signal transduction cascade of endogenous cardioprotection [23].

NO is also involved in cardioprotective signaling [24, 69] and NO’s role in post-translational modification of proteins has been increasingly recognized in recent years [59, 63, 70], especially during the endogenous cardioprotective interventions [31]. SNO has been shown to play an important role in the regulation of metabolism and myocardial key proteins like the L-type Ca^2+^ channel [71], the sarcoplasmic reticulum Ca^2+^-ATPase [69], the sarcoplasmic Ca^2+^ release channel [18, 78], and guanylyl cyclase [58]. Furthermore, it alters the activity of mitochondrial proteins such as cytochrome *c* oxidase [81], F_1_F_0_ATPase, and mitochondrial respiratory chain complex 1 [69]. Application of a selective mitochondrial SNO donor inhibits mitochondrial respiratory chain complex 1 activity during the first 5 min of reperfusion in ischemic tissue thereby protecting from oxidative damage by decreasing ROS production [8].

Since SNO regulates Cx43 at the sarcolemma, the question arises whether or not SNO regulates mtCx43 as well. In the present study, we investigated if SNO modifies mtCx43 and influences mitochondrial permeability, ion fluxes, and ROS generation.

## Materials and methods

### Animals

All rodents were treated according to the Guide for the Care and Use of Laboratory Animals published by the US National Institutes of Health (NIH publication no. 85-23, revised 1996) and approved by the Institutional Laboratory Animal Care and Use Committee of the NIH, Bethesda, MD, USA or according to the European Convention for Protection of Vertebrate Animals Used for Experimental and Other Scientific Purposes (Council of Europe Treaty Series No. 123). For Langendorff heart perfusion experiments male Sprague–Dawley rats (190–210 g and 7–8 weeks old) were anesthetized with pentobarbital and anti-coagulated with heparin. Male Wistar–Janvier rats (190–210 g and 8–10 weeks old) were used for mitochondrial permeability experiments and were anesthetized with 2.5 % v/v isoflurane. Male C57BL/6J mice, 11–14 weeks of age with a body weight ranging from 25 to 35 g, were anesthetized by intraperitoneal (i.p.) injection of ketamine (45 mg/kg) and xylazine (Rompun, 10 mg/kg).

### Rat heart perfusion protocols

Excised rat hearts were placed in ice-cold Krebs-Henseleit buffer (120 mmol NaCl, 11 mmol d-glucose, 25 mmol NaHCO_3_, 1.75 mmol CaCl_2_, 4.7 mmol KCl, 1.2 mmol MgSO_4_, and 1.2 mmol KH_2_PO_4_) and the hearts were Langendorff perfused in retrograde fashion with oxygenated Krebs–Henseleit buffer (95 % O_2_/5 % CO_2_, pH 7.4) at a constant pressure of 100 cm water at 37 °C. Hearts were randomly assigned to either a control group perfused for 40 min under normoxic conditions or an ischemic preconditioned (IPC) group where the hearts were perfused for 20 min under normoxic conditions followed by four cycles of 5 min ischemia and 5 min reperfusion [35, 69]. Perfusion was performed in the dark to prevent breakdown of SNO modifications. Control experiments were also performed by daylight confirming light sensitivity of SNO modifications.

### Nitrite-induced SNO of mtCx43

C57BL/6 mice were anesthetized by intraperitoneal injection of ketamine (45 mg/kg) and xylazine (Rompun 10 mg/kg). A tracheal tube was inserted for mechanical ventilation, which was performed according to the individual body weight at a tidal volume of 2.1–2.5 ml and a respiratory rate of 140 breaths per minute. The mice were supplemented with 100 % oxygen via a rodent ventilator (Minivent) side port. Pharmacological preconditioning was induced by injection of 48 nmol sodium nitrite into the cavity of the left ventricle, whereas the control group received an equal volume of 50 µl physiological saline. The mouse chests were opened through a midline sternotomy. Subsequent mouse hearts were excised, mitochondria were isolated, and SNO of mtCx43 was quantified by using the modified biotin switch method. For details of the preparation as well as the extent of infarct size reduction achieved by this protocol see Rassaf et al. [46].

### Isolation of mitochondria

All procedures were performed at 4 °C to maintain mitochondrial integrity. To prevent light-induced SNO breakdown, samples were kept in the dark during the isolation process. SSM were isolated from left ventricles of male rats as described previously [1]. For functional analysis, left rat ventricles were minced in isolation buffer containing 250 mmol sucrose, 10 mmol HEPES, 1 mmol EDTA, and 0.5 % BSA (pH 7.4 adjusted with Tris base), homogenized with an Ultra Turrax and centrifuged at 800*g* for 10 min. The resulting supernatant was centrifuged for 10 min at 10,780*g* and the pellet was resuspended in BSA-free isolation buffer and centrifuged twice at 7,650*g* for 5 min.

Interfibrillar mitochondria (IFM), which lack Cx43, were used as negative controls and isolated as previously described [5]. Minced rat left ventricles were weighed, homogenized and centrifuged at 800*g* for 10 min. The resulting pellet was resuspended in 5 ml isolation buffer additional containing 0.5 % BSA and Nargase (8 U/g) and incubated on ice for 1 min. The tissue was homogenized and centrifuged at 800*g* for 10 min and the supernatant was centrifuged for 10 min at 10,780*g*. The resulting pellet was resuspended in BSA free isolation buffer and centrifuged twice at 7,650*g* for 5 min.

For quantitative SNO analysis, a BSA-free isolation buffer with SNO modifications protecting agents was used containing 250 mmol sucrose, 10 mmol HEPES, 1 mmol EDTA, TRIS-base (pH 7.4), 0.1 mmol neocuproine, EDTA free complete protease inhibitor (Roche Diagnostics, Grenzach, Germany), and phosphatase inhibitor (Roche Diagnostics, Grenzach, Germany). The mitochondria were isolated as described above and then layered on a 30 % Percoll gradient and centrifuged for 30 min at 35,000*g*. This resulted in a lower fraction containing pure SSM and an upper fraction containing cell debris. The lower fraction was collected and washed three times with isolation buffer by centrifuged at 10,200*g* for 5 min.

Protein concentrations were determined by the Lowry assay using BSA as a standard. For quantitative analysis, the purity of isolated mitochondria was validated by Western blot analysis confirming the absence of non-mitochondrial cellular proteins and the enrichment of mitochondrial proteins. Mitochondria were stored at −80 °C.

### Dye permeation experiments

Dye permeation experiments were performed according to Miro-Casas et al. [36]. Freshly isolated mitochondria were pelleted by centrifugation at 10,200*g* for 3 min at 4 °C and resuspended at a concentration of 400 µg/ml in isosmotic succinate buffer (150 mmol KCl, 7 mmol NaCl, 2 mmol KH_2_PO_4_, 1 mmol MgCl_2_, 6 mmol MOPS, pH 7.2, 6 mmol succinate, 0.25 ADP, and 0.5 µmol rotenone). Mitochondria were assigned to six groups and were either supplemented with 1 or 25 µmol of the hemichannel blocker carbenoxolone (CBX; Sigma-Aldrich, Heidenheim, Germany), 0.5 mmol NO donor *S*-nitroso-*N*-acetyl-dl-penicillamine (SNAP; Life Technologies, Darmstadt, Germany), 1 mmol of NO donor *S*-nitrosoglutathione (GSNO; Santa Cruz, Heidelberg, Germany), a combination of NO donor and CBX, or 5 µl dimethyl sulfoxide (DMSO; used as solvent, Sigma-Aldrich, Heidenheim, Germany). Additional mitochondria from Langendorff perfused hearts receiving either IPC or control perfusion were assigned to dye permeation experiments. As negative control, mitochondria were exposed to UV light for 5 min to remove light sensitive SNO modifications.

After a 20 min incubation period at 25 °C and 650 rpm, 50 µmol/l of the Cx43 hemichannel-permeable dye Lucifer Yellow CH dilithium salt (LY; Sigma-Aldrich, Heidenheim, Germany) and 25 µg/ml of the hemichannel-impermeable dye Rhodamine B isothicyanate-dextran 10S (RITC-dextran; Sigma-Aldrich, Heidenheim, Germany) were added and samples were incubated for 25 min at 25 °C and 650 rpm. Subsequently, mitochondria were washed and resuspended in 200 µl succinate buffer. Fluorescence of LY (*λ*_ex_ 430 nm, *λ*_em_ 535 nm) and RITC-dextran (*λ*_ex_ 545 nm, *λ*_em_ 600 nm) was measured by a 96-microplate fluorometer (Cary Eclipse spectrophotometer, Varian, Mulgrave, Australia) at high sensitivity [36]. As a negative control, experiments were performed with ultrasound treated (20 s, amplitude 50 %) mitochondria to exclude interference of dye and membrane fragments, as well as with IFM to confirm Cx43 hemichannel-specific effects.

### Mitochondrial K^+^ uptake

Experiments for measuring velocity of mitochondrial K^+^ influx were modified from Miro-Casas et al. [36]. Freshly isolated mitochondria were resuspended at a concentration of 400 µg/ml in isolation buffer. Either 1, 10, or 25 µmol CBX, 0.5 mmol SNAP, the combination of both, or 5 µl DMSO was added to mitochondria and incubated for 20 min at 25 °C and mixed at 650 rpm. The experiments were repeated with the use of 1 mmol physiologically relevant NO donor GSNO instead of SNAP. Subsequently, mitochondria were loaded with 10 µmol/l acetoxymethyl of potassium-binding benzofuran isophthalate (PBFI; Life Technologies, Darmstadt, Germany) for 10 min at 25 °C and 650 rpm. K^+^ depletion from the mitochondrial matrix was achieved by adding 3 volumes of tetraethylammonium (TEA) buffer (120 mmol TEA-Cl, 10 mmol HEPES, 10 mmol succinate, 5 mmol Na_2_HPO_4_, 0.1 mmol EGTA, 0.5 mmol MgCl_2_, 5 µmol rotenone, and 0.67 µmol oligomycin, pH 7.2), which replaces K^+^ in the mitochondrial matrix. Subsequently, mitochondria were washed, sedimented at 10,200*g* and 4 °C for 3 min, and resuspended in 30 µl isolation buffer. The kinetics of mitochondrial K^+^ uptake were measured after a KCl pulse of 140 mmol at alternated excitations at 340/380 nm and emission at 500 nm by a fluorometer (Cary Eclipse spectrophometer; Varian, Mulgrave, Australia) in 2 ml isolation buffer at medium sensitivity. To measure the K^+^ permeability for the Cx43 hemichannel only, during measurements, mitochondrial permeability transition pore (MPTP) opening was blocked by adding 1 µmol cyclosporine A (CsA; Sigma-Aldrich, Heidenheim, Germany) [22], the proton channel of the ATP-synthase was blocked with 1 µg/ml oligomycin (Sigma-Aldrich, Heidenheim, Germany), and opening of ATP-dependent potassium channels was blocked by 5 µmol glibenclamide (Sigma-Aldrich, Heidenheim, Germany) [62, 72]. The experiments were repeated without these inhibitors to investigate the influence of NO donors on the K^+^ influx under more physiological conditions. The K^+^ influx, the first 2 s after the KCl-pulse, was determined by the increase of the PBFI fluorescence ratio of 340/380 nm per second of the six different treated groups. As a positive control, K^+^ influx was determined after addition of 5 nmol valinomycin, which is highly sensitive to sodium (Na^+^) and K^+^ and functions as a K^+^-specific transporter across membranes. To confirm a Cx43 specific effect, experiments were also performed with IFM instead of SSM as Cx43 negative controls.

### Mitochondrial Na^+ ^uptake

Mitochondria were treated either with 25 µmol CBX, 0.5 mmol SNAP, 1 mmol GSNO, the combination of NO donor and CBX, or 5 µl DMSO and then loaded with 10 µmol/l acetoxymethyl of sodium-binding benzofur (SBFI; Life Technologies, Darmstadt, Germany) instead of using PBFI. Sodium depletion was then performed with tetramethylammonium (TMA) buffer (120 mmol TMA-Cl, 10 mmol HEPES, 10 mmol succinate, 5 mmol KH_2_PO_4_, 0.1 mmol EGTA, 0.5 mmol MgCl_2_, 5 µmol rotenone, and 0.67 µmol oligomycin, pH 7.2). After subsequent washing, the kinetics of mitochondrial Na^+^ uptake were measured after a NaCl pulse of 10 and 140 mmol at alternated excitations at 340/380 nm and emission at 500 nm by a fluorometer (Cary Eclipse spectrophotometer; Varian, Mulgrave, Australia). This was performed in 2 ml isolation buffer as well as in 2 ml succinate buffer supplemented with inhibitors for MPTP, ATP-synthase, and ATP-dependent potassium channels as described above at high sensitivity. Na^+^ influx of the first 2 s after the NaCl-pulses was determined by the increase of the SBFI fluorescence ratio of 340/380 nm per second of the six different treated groups. As a positive control, Na^+^ influx was determined after addition of 5 nmol gramicidin, which has a similar function as valinomycin and forms a channel for K^+^ and Na^+^, but does not interfere with SBFI dye as does valinomycin.

### ROS production

One mg of freshly isolated SSM or IFM were added to 2 ml incubation buffer (150 mmol KCl, 7 mmol NaCl, 2 mmol KH_2_PO_4_, 1 mmol MgCl_2_, and 6 mmol MOPS, pH 7.4) containing 5 mmol glutamate and 2.5 mmol malate as a substrate, 1 U/ml horseradish peroxidase (HRP; Roche Diagnostics, Grenzach, Germany), and 50 µmol Amplex UltraRed reagent (Thermo Fisher Scientific Inc., Darmstadt, Germany). Amplex UltraRed is a fluorogenic substrate for HRP that reacts with hydrogen peroxide (H_2_O_2_). For estimating the NO-mediated influence of mtCx43-dependent ROS production either 1 or 25 µmol CBX, 0.5 mmol SNAP, 1 mmol, 1 µmol, or 48 nmol GSNO, a combination of NO donor and CBX, or 20 µl dH_2_O was added 30 s before the measurement was started. In previous studies, NO donors—like SNAP or GSNO—were given at concentrations from 10 μmol to 1 mmol. Therefore, in the present study we used mmol concentrations but lowered them down to nmol concentrations. Additional experiments were performed with Cx43 inhibition by the connexin mimetic peptide Gap26 [12] either in the presence of 1 mmol GSNO or not. Experiments were repeated under respiratory chain uncoupling conditions induced by 30 nmol carbonyl cyanide *p*-(tri-fluromethoxy)phenyl-hydrazone (FCCP) and with 2 µmol of the respiratory chain complex 1 inhibitor rotenone. Analysis of ROS formation was performed by a fluorometer (Cary Eclipse spectrophometer; Varian, Mulgrave, Australia) at an extinction and emission wavelength of 568/581 nm. Mitochondrial H_2_O_2_ production was measured for 4 min and the increase of H_2_O_2_ was expressed in nmol/min/mg protein by comparing the data to a standard curve. After 4 min of measurement, 2 µg/ml of mitochondrial complex III inhibitor antimycin A (Sigma-Aldrich, Heidenheim, Germany) was added for inducing mitochondrial ROS overproduction, which served as a positive control.

### Labeling and precipitation of SNO modified proteins

A modified biotin switch method was used for labeling and quantification of SNO residues as previously described [27]. SSM protein samples (250 µg) and controls (250 µg IFM, and 250 µg right ventricle of rodent hearts) were diluted in HEN buffer (250 mmol HEPES-NaOH pH 7.7, 1 mmol EDTA, and 0.1 mmol neocuproine), supplemented with EDTA free complete protease inhibitor tablet (Roche Diagnostics, Indianapolis, IN, USA), phosphatase inhibitor (Roche Diagnostics, Grenzach, Germany or Indianapolis, IN, USA) and 2.5 % SDS (wt/vol). To block free thiols, 50 mmol *N*-ethylmaleimide (NEM; Sigma-Aldrich, St. Louis, MO, USA or Heidenheim, Germany) was used. After incubation for 20 min at 50 °C with gentle mixing every 5 min, free thiols were labeled with NEM and could not be modified. This procedure was stopped by removing NEM via cold acetone precipitation (−20 °C). The samples were then resuspended in HEN buffer with 1 % SDS (wt/vol) containing 1 mmol ascorbate (Sigma-Aldrich, St. Louis, MO, USA) for reduction of SNO modified cysteine residues. Reduced SNO groups were labeled with *N*-(biotinoyl)-*N*-(iodoacetyl)ethylenediamine (BIAM; Sigma-Aldrich, St. Louis, MO, USA or Heidenheim, Germany). Prior to incubating samples with streptavidin-agarose beads (Sigma-Aldrich, St. Louis, MO, USA) for precipitation of SNO modified proteins, 2 µl of loading control was taken. Precipitation was performed overnight with rotation at 4 °C in the dark. Samples were washed three times with HEN buffer, eluted in 30 µl sample buffer with 10 M urea and heated at 95 °C for 5 min. Specificity of the biotin switch method was proven by adding 10 or 100 mmol DTT before eluting the sample, which breaks the disulfide bound between the thiol group and biotin. Western blot analysis was subsequently performed.

### Effects of NO donors on mtCx43 phosphorylation

The influence of NO donors on the phosphorylation status of mtCx43 was analyzed. Mitochondria were incubated either with 1 µmol CBX, 0.5 mmol SNAP, 1 mmol GSNO, the combination of a NO donor and CBX, or 5 µl DMSO in isolation buffer. Subsequent mitochondrial purity was achieved by Percoll gradient centrifugation as described above. Phosphorylation of Cx43 was then analyzed and quantified by using a phospho-Cx43 specific antibody and Western Blot analyses.

### Quantification of SNO-modified mtCx43, analysis of Cx43 phosphorylation, and purity control of mitochondrial preparation by western blot analysis

Right ventricles (RV) of rat or mouse hearts were minced and used as positive controls. All controls were diluted with RIPA lysis buffer (Invitrogen, Grand Island, NY, USA) and centrifuged at 13,000*g* for 10 min at 4 °C. Protein concentration of supernatants was estimated using the Bradford assay or Lowry assay using BSA as a standard. Both mitochondrial samples and controls were separated by electrophoresis on 10 % Bis–Tris SDS-Gels (NextGen, Ann Arbor, MI, USA) and transferred to nitrocellulose membranes. Protein transfer was controlled by membrane staining with Ponceau S. After blocking, membranes were incubated with primary antibodies. The corresponding IgG HRP-conjugate combined with chemiluminescent substrate (GE Healthcare Life Sciences, Freiburg, Germany) or corresponding DyLight maleimide sulfhydryl-reactive dyes (Pierce Biotechnology, Rockford, IL, USA) combined with fluorescence emission at 700 nm were used as secondary antibodies and scanned on a Typhoon 9400 variable mode imager (GE Healthcare Lifesciences, Piscataway, NJ, USA).

### Antibodies

The following antibodies were used in this study: rabbit polyclonal anti-rat total connexin43 (Invitrogen, Carlsbad, CA, USA), mouse monoclonal anti-rat sodium/potassium (Na^+^/K^+^)-ATPase (Upstate, Waltham, MA, USA), mouse monoclonal anti-dog sarcoplasmic calcium (SERCA2)-ATPase (Sigma-Aldrich, St. Louis, MO, USA), rabbit monoclonal anti-human histone deacetylase 2 (HDAC2; Abcam, Cambridge, UK), mouse monoclonal anti-rabbit GAPDH (Hytest, Turku, Finland), rabbit polyclonal anti-human translocase of the outer membrane 20 (Tom20; Santa Cruz, CA, USA), rabbit polyclonal anti-human voltage-dependent anion channel (VDAC) (Abcam, Cambridge, UK), and rabbit polyclonal anti-human manganese superoxide dismutase (MnSOD, Upstate, Lake Placid, NY, USA). The phospho-Cx43 antibody Cx43-pS368 (#3511, Cell Signaling, Leiden, The Netherlands) was used for analyses of Cx43 phosphorylation.

### Statistics

Data are presented as mean ± SEM. Normal distribution of data was analyzed using a non-parametric Kolmogorov–Smirnov test. Unpaired Student’s *t* test was used between the two groups of IPC and control mitochondria of Western blot data and dye permeation data to determine differences in mean values. Data of LY, K^+^ uptake and ROS formation were compared by two-way repeated measures ANOVA and Fisher’s LSD. Statistical significance was determined at *p* < 0.05.

## Results

### Effects of SNO modified Cx43 on mitochondrial uptake of LY

**Fig. 1:**
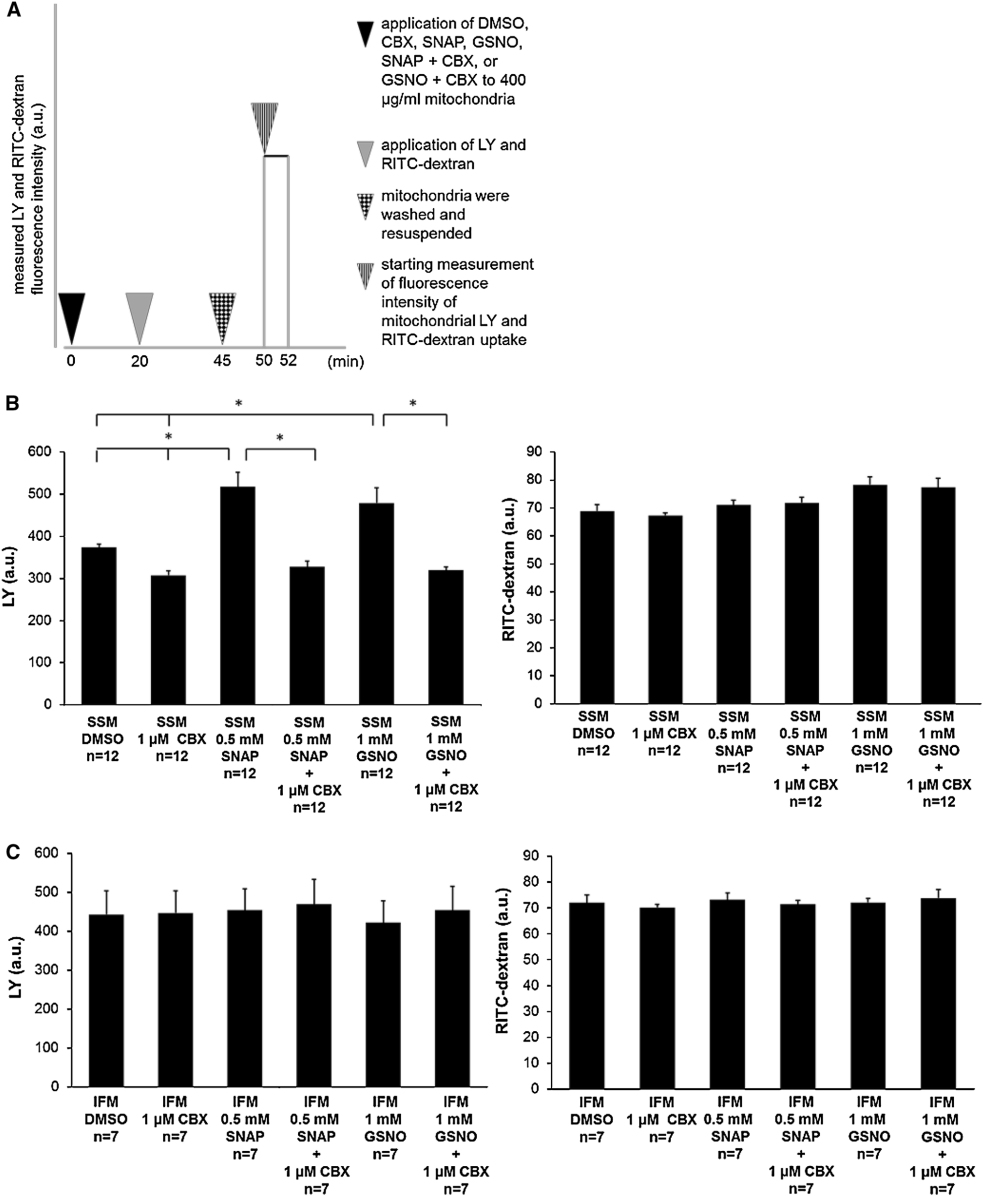
Measurements of mitochondrial permeability. SSM and IFM were treated either with carbenoxolone (CBX), *S*-nitroso-*N*-acetyl-dl-penicillamine (SNAP), *S*-nitrosoglutathione (GSNO), a combination of NO donor and hemichannel blocker (SNAP + CBX; GSNO + CBX), or dimethyl sulfoxide (DMSO; used as solvent). Dye uptake of hemichannel permeable LY and hemichannel impermeable dye RITC-dextran was measured and expressed as arbitrary units of fluorescence (**a**). Data of LY (*left panel*) and RITC uptake (*right panel*) are shown as mean ± SEM of 7–12 replicates per group from intact SSM (**b**) and IFM (**c**) from rat left ventricles. *Asterisk* (*p* < 0.05) indicates significant differences between marked groups

Rat mitochondria were treated separately either with 5 µl DMSO, 1 µmol or 25 µmol carbenoxolone (CBX), 0.5 mmol SNAP, 1 mmol GSNO, or a combination of a NO donor and CBX. Fluorescence analyses were performed after exposure to 50 µmol LY and 25 µg/ml RITC-dextran (Fig. 1a). Mitochondrial LY fluorescence intensity was significantly increased after application of NO donor SNAP compared to DMSO control by 38.4 ± 7.1 % (*n* = 12, *p* < 0.05). The NO-mediated increased mitochondrial permeability for LY was verified by application of NO donor GSNO instead of SNAP. GSNO application increased LY uptake by 28.1 ± 7.4 % (*n* = 12, *p* < 0.05) compared to control. The NO-mediated increase in LY fluorescence was abolished in SSM incubated with the Cx43 hemichannel blocker, CBX. CBX at a concentration of 1 µmol decreased LY uptake by 17.7 ± 0.8 % compared to DMSO control (*n* = 12, *p* < 0.05), by 36.7 ± 4 % (SNAP + CBX versus SNAP; *n* = 12, *p* < 0.05) compared to SNAP, and by 33.4 ± 5.6 % (GSNO + CBX versus GSNO; *n* = 12, *p* < 0.05) compared to GSNO-treated mitochondria. Increasing the CBX concentration up to 25 µmol had a similar effect on mitochondrial permeability. On the other hand, the fluorescence intensity of the hemichannel impermeant dye RITC-dextran showed no difference throughout the different protocols (*n* = 12, *p* = ns) (Fig. 1b).

Additionally, the experiments were repeated with IFM, a population of mitochondria which do not contain Cx43, to validate that the effect of increased permeability caused by SNO is specific for Cx43 hemichannels containing mitochondria. IFM did not show a difference in dye uptake between all treatment groups (*n* = 7, *p* = ns) (Fig. 1c). Cross reactions of LY with membrane fragments were excluded by repeating the experiments following ultrasound treatment, to rupture the mitochondria. These studies with ultrasound showed no significant alteration in LY uptake by any of the treatments (Supplemental Figure 1, *n* = 7, *p* = ns). Furthermore, fluorescence intensity of RITC-dextran did not differ with the different protocols (Supplemental Figure 1, *n* = 7, *p* = ns).

**Fig. 2:**
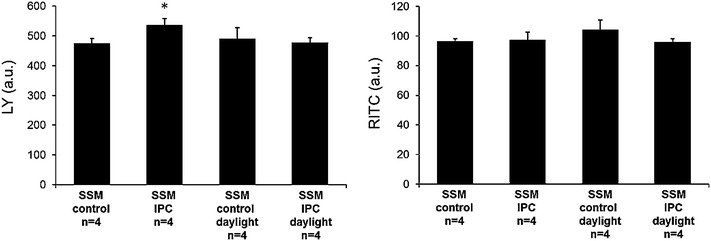
Mitochondrial LY dye uptake following Langendorff perfusion of rat hearts. The dye uptake of SSM from left ventricles of Langendorff perfused rat hearts receiving IPC or control perfusion was measured. The mitochondrial dye uptake of hemichannel permeable LY (*left panel*) and hemichannel impermeable RITC-dextran (*right panel*) was measured and expressed as arbitrary units of fluorescence. As a control, mitochondria were additionally exposed to daylight before incubation with dyes confirming light sensitivity of SNO protein modifications. Data are shown as mean ± SEM of four replicates per group. *Asterisk* (*p* < 0.05) indicates significant differences between groups

The permeability for LY and RITC-dextran was also analyzed in preconditioned mitochondria and mitochondria from control perfused rat hearts. The mitochondria from IPC rat hearts had a 13.1 ± 4.6 % (Fig. 2, left panel, *n* = 4, *p* < 0.05) higher LY dye uptake compared to mitochondria from control perfused hearts. However, the RITC-dextran fluorescence intensity did not differ between the groups (*n* = 4, *p* < 0.05). In addition, after exposure to UV light there was no detectable difference in dye uptake between IPC and control mitochondria (Fig. 2, right panel, *n* = 4, *p* < 0.05).

### Mitochondrial K^+^/Na^+^ uptake

**Fig. 3:**
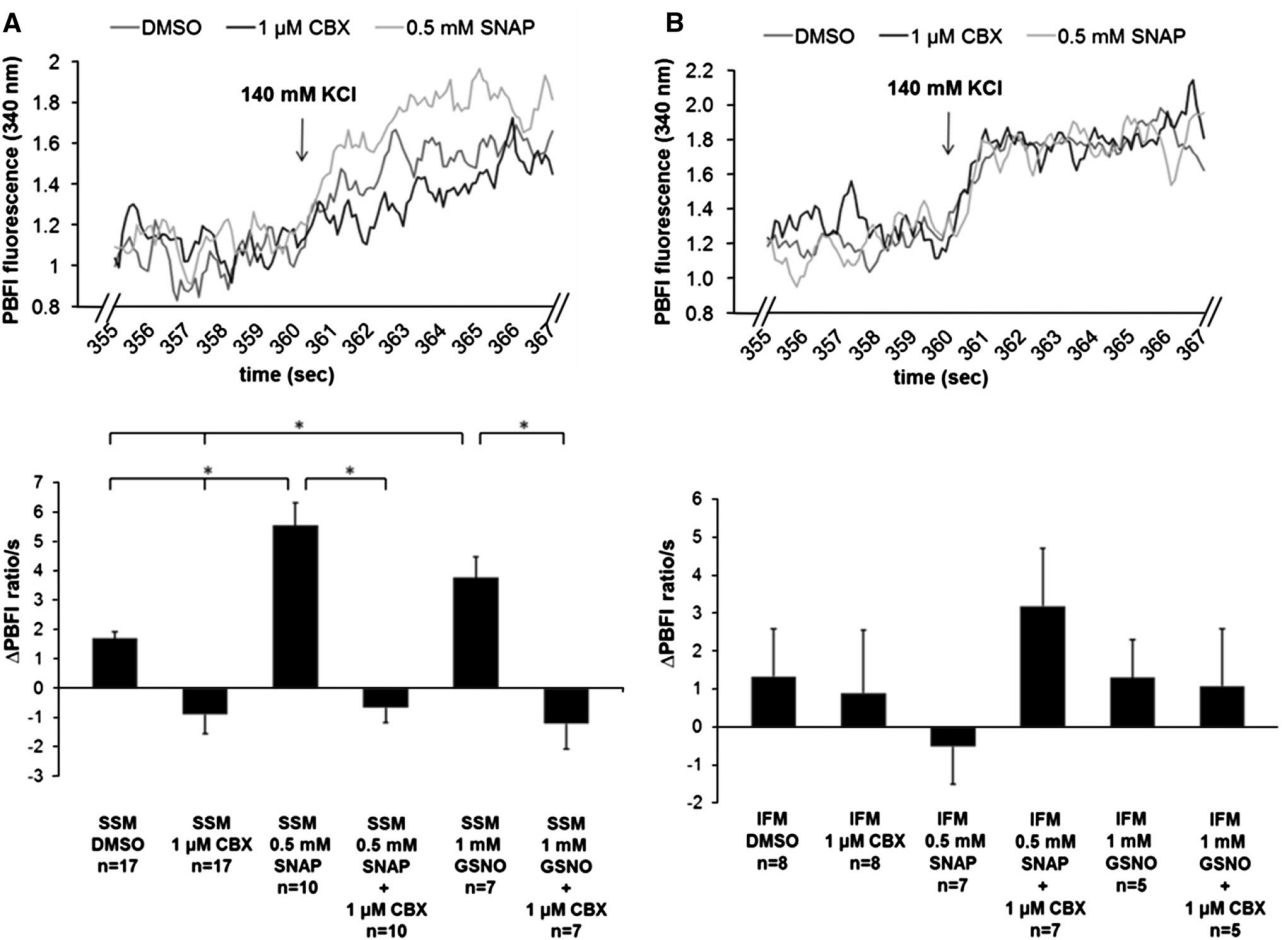
Mitochondrial K^+^ permeability. Analysis of K^+^ influx was measured in SSM (**a**) and IFM (**b**) from the LV of rat hearts with inhibition of MPTP, ATP-synthase, and ATP-dependent potassium channels. Mitochondria were either treated with carbenoxolone (CBX), *S*-nitroso-*N*-acetyl-dl-penicillamine (SNAP), *S*-nitrosoglutathione (GSNO) a combination of NO donor and hemichannel blocker (SNAP + CBX; GSNO + CBX), or dimethyl sulfoxide (DMSO; used as solvent). Oligomycin, glibenclamide, and cyclosporine were present during the entire experiment to measure K^+^ influx exclusively via Cx43 hemichannels. The rate of PBFI fluorescence ratio (340/380 nm) change from mitochondria in different treatments was estimated for the initial 2 s after the addition of a 140 mmol KCl pulse. Data correspond to mean ± SEM of 7–17 replicates per group. *Asterisk* (*p* < 0.05) indicates significant differences between marked groups

Mitochondria were incubated separately either with 1, 10, or 25 µmol CBX, 0.5 mmol SNAP, 1 mmol GSNO, a combination of NO donor and CBX, or 5 µl DMSO used as a vehicle. The velocity of K^+^ influx into the matrix of mitochondria from rat left ventricles was estimated by measuring the increase of the 340/380 nm fluorescence intensity ratio for 2 s after addition of a 140 mmol KCl pulse. To measure the K^+^ permeability through Cx43 hemichannels, other mitochondrial channels including ATP-dependent potassium channels, ATP-synthase, and MPTP were blocked with glibenclamide, oligomycin, and cyclosporine A. Mitochondria treated with NO donors showed a significant higher refilling rate of K^+^. The velocity of K^+^ uptake was 227.9 ± 30.1 % higher in SNAP-treated mitochondria compared to DMSO control (*n* = 10, *p* < 0.05). Application of the NO donor GSNO increased the velocity of K^+^ influx by 122.6 ± 28.1 % (*n* = 7, *p* < 0.05) compared to control. The NO-mediated increases of mitochondrial K^+^ influx by SNAP and GSNO were significantly blocked by CBX at a concentration of 1 µmol. With CBX application, velocity of K^+^ influx decreased by 153.2 ± 24.4 % (*n* = 17, *p* < 0.05) compared to DMSO control, decreased by 112.1 ± 4.5 % in SNAP (SNAP + CBX versus SNAP; *n* = 7, *p* < 0.05), and decreased by 170 ± 4.3 % (GSNO + CBX versus GSNO; *n* = 7, *p* < 0.05) compared to GSNO treated mitochondria (Fig. 3a). Again, IFM did not show alterations of K^+^ influx in response to NO donors or CBX (*n* = 7, *p* = ns) (Fig. 3b).

**Fig. 4:**
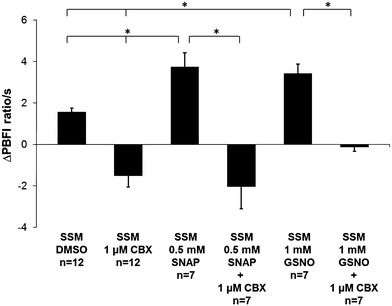
Mitochondrial K^+^ permeability. Analysis of K^+^ influx under physiological conditions was measured without adding additional mitochondrial channel blockers to SSM from LVs of rat hearts. Mitochondria were either treated with carbenoxolone (CBX), *S*-nitroso-*N*-acetyl-dl-penicillamine (SNAP), *S*-nitrosoglutathione (GSNO), a combination of NO donor and hemichannel blocker (SNAP + CBX; GSNO + CBX), or dimethyl sulfoxide (DMSO; used as solvent). The increase in the PBFI fluorescence ratio of 340/380 nm (arbitrary units) was measured for 2 s after an initial KCl pulse of 140 mmol. Data correspond to mean ± SEM of 7–12 replicates per group. *Asterisk* (*p* < 0.05) indicates significant differences between marked groups

SNO-mediated K^+^ influx through Cx43 hemichannels was also measured without additional mitochondrial channel blockers. The data showed a NO-mediated increase of K^+^ influx, which was somewhat weaker with SNAP versus DMSO (138.9 ± 30.7 %, *n* = 7, *p* < 0.05) compared with SNAP versus DMSO with additional mitochondrial channel blockers. GSNO also increased K^+^ permeability compared to control (119.7 ± 16 %, *n* = 7, *p* < 0.05) as was the case in experiments where the K^+^ permeability for Cx43 hemichannels was measured in the presence of inhibitors. The NO-mediated increase of K^+^ influx was blocked by CBX (154.6 ± 10.1 % for 0.5 mmol SNAP with 1 µmol CBX and 103.8 ± 25.2 % for 1 mmol GSNO with 1 µmol CBX; *n* = 7, *p* < 0.05) (Fig. 4).

Permeability for Na^+^ was measured using the Na^+ ^ binding benzoflur SBFI. Only small, non-significant changes in Na^+ ^fluxes were measured (Supplemental Figure 2, *n* = 6, *p* = ns) suggesting that mitochondrial Cx43 hemichannels have certain ion selectivity. Experimental set up function was proven by application of gramicidin, which forms a channel for Na^+^ in the mitochondrial membrane. Application of gramicidin leads to Na^+^ influx, which did not differ between groups.

### ROS production

**Fig. 5:**
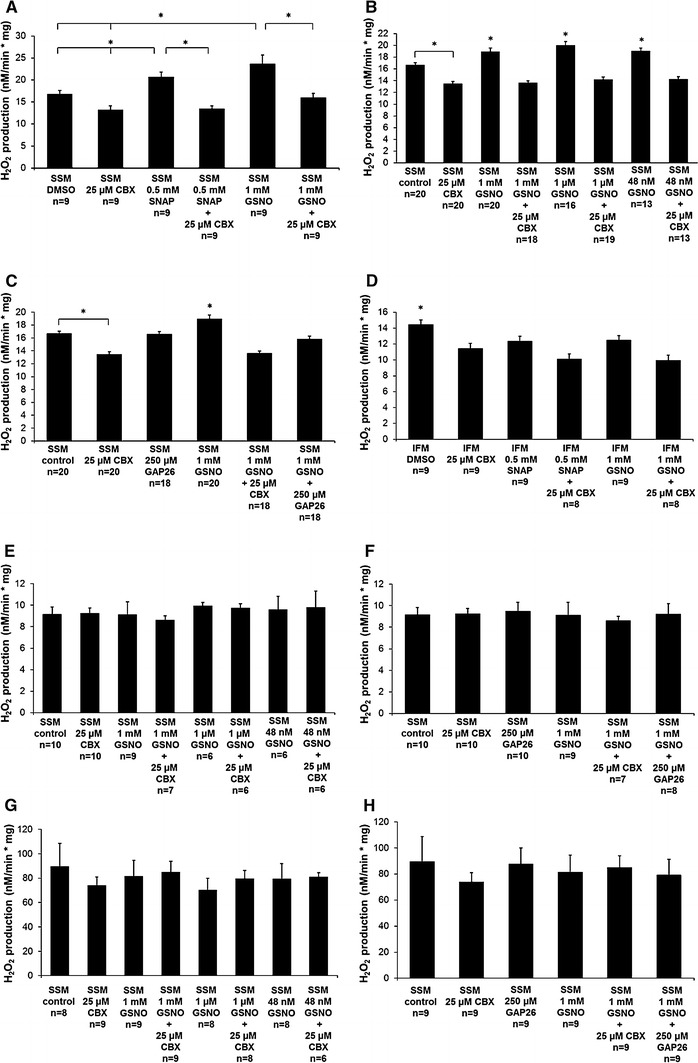
Quantification of ROS formation was performed on freshly isolated SSM (**a**–**c**) and IFM (**d**) treated with carbenoxolone (CBX), *S*-nitroso-*N*-acetyl-dl-penicillamine (SNAP), *S*-nitrosoglutathione (GSNO), a combination of a NO donor and hemichannel blocker (SNAP + CBX; GSNO + CBX), or dH_2_O (**a**). Additionally, ROS formation was measured with lower concentrations (1 µmol and 48 nmol) of GSNO (**b**) and was measured after application of 250 µmol Cx43 mimetic peptide Gap26 (**c**). Mitochondrial H_2_O_2_ production was measured during ADP stimulated complex 1 respiration for 4 min and the increase of H_2_O_2_ was expressed in nmol/min/mg protein by comparing the data to a standard curve. As a control, respiration chain uncoupler FCCP (**e**, **f**) and respiration chain complex 1 inhibitor rotenone (**g**, **h**) were applied to ROS measurements. Data correspond to mean ± SEM of 6–20 replicates per group. *Asterisk* (*p* < 0.05) indicates significant differences between marked groups

Studies suggest that endothelial NOS docks to the mitochondrial outer membrane producing local NO [16]. Therefore, whether or not SNO of mitochondrial Cx43 influences mitochondrial ROS formation is of interest. ROS production of mitochondria was analyzed using 1, or 25 µmol CBX, Gap26, 0.5 mmol SNAP, 1 mmol, 1 µmol, or 50 nmol GSNO, a combination of a NO donor and CBX or Gap26, or 20 µl dH_2_O. During ADP stimulated complex 1 respiration, ROS production was increased by application of the NO donor SNAP (22.9 ± 1.8 %, *n* = 9, *p* < 0.05) compared to control and by using GSNO (40.6 ± 7.1 %, *n* = 9, *p* < 0.05) compared to control. These increases were abolished by 25 µmol CBX (Fig. 5a). Concentrations of 1 µmol and 48 nmol GSNO increased ROS production by 20 ± 3.7 % (*n* = 16, *p* < 0.05) and 14.3 ± 2.8 % (*n* = 13, *p* < 0.05) respectively compared to control (Fig. 5b). The Cx43 mimetic peptide, Gap26 is known to inhibit GJs and non-junctional hemichannels [79]. Application of the Cx43 mimetic peptide Gap26 decreased ROS formation by 16.5 ± 2.6 % (*n* = 19, *p* < 0.05) compared to GSNO treated mitochondria, but ROS formation did not differ compared to control treated mitochondria (Fig. 5c). In IFM, the NO donors decreased ROS production. SNAP decreased ROS formation (14.4 ± 4 %, *n* = 9, *p* < 0.05) compared to control as did GSNO (13.8 ± 4 %, *n* = 9, *p* < 0.05) compared to control. CBX had no significant inhibitory effect on ROS production with ADP stimulated complex 1 respiration (*n* = 9, *p* = ns) (Fig. 5d).

Additionally, ROS measurements were performed using the respiratory chain uncoupling agent FCCP and the respiratory chain inhibitor rotenone. FCCP significantly decreased ROS formation by 45.2 ± 3.9 % (control vs. control FCCP; *n* = 10, *p* < 0.05), whereas ROS generation did not differ between the different treatment groups (Fig. 5e, f). In contrast, the respiratory chain complex 1 inhibitor rotenone increased ROS formation by 436.3 ± 113.6 % (control vs. control rotenone, *n* = 8, *p* < 0.05). However, ROS formation in the presence of rotenone did not differ between groups (Fig. 5g, h).

### Quantification of SNO modified mtCx43 after NO donor application and IPC

**Fig. 6:**
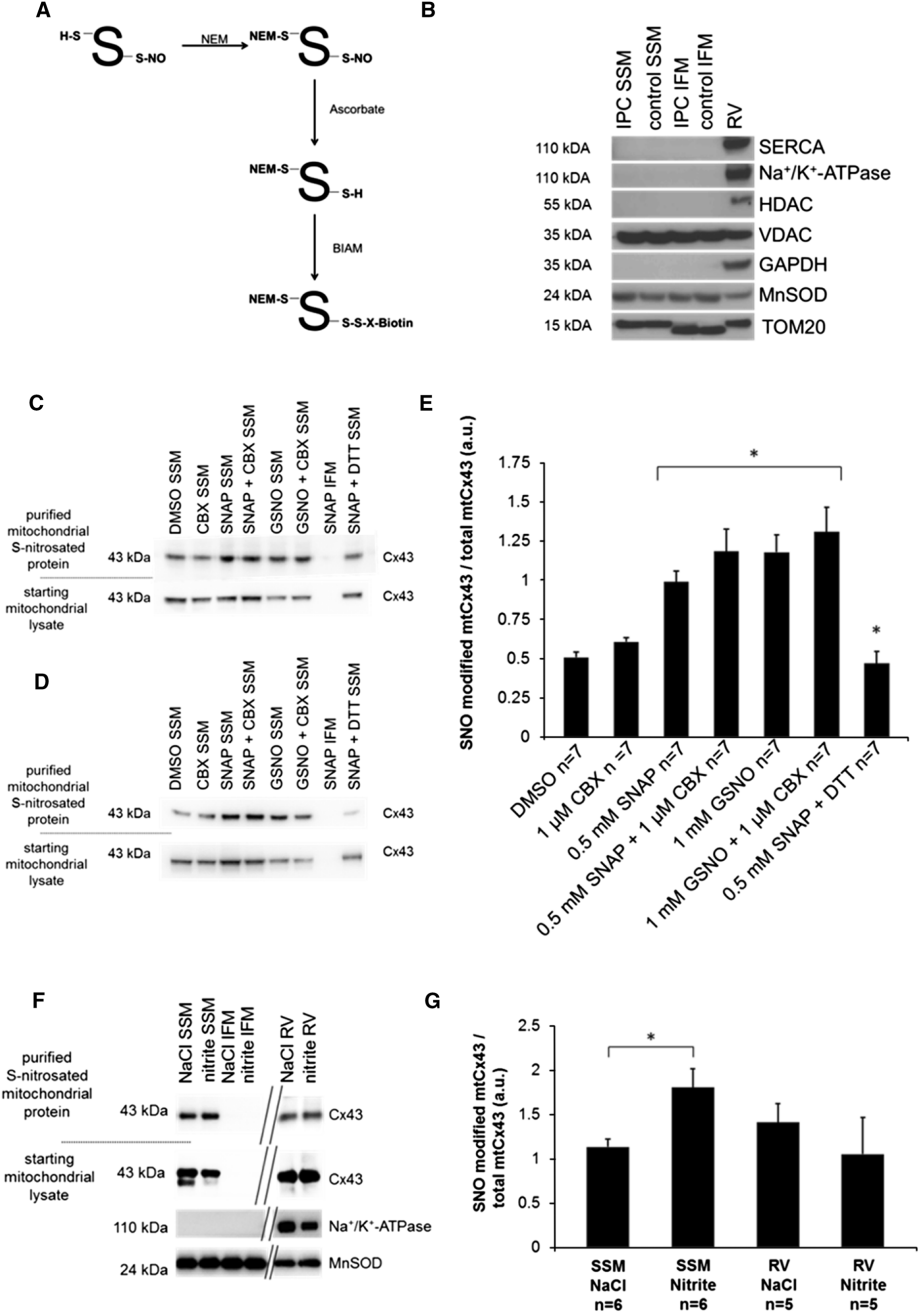
SNO quantification of mtCx43 for NO treated mitochondria. SNO quantification was performed on mitochondria treated either with carbenoxolone (CBX), *S*-nitroso-*N*-acetyl-dl-penicillamine (SNAP), *S*-nitrosoglutathione (GSNO), a combination of NO donor and hemichannel blocker (SNAP + CBX; GSNO + CBX), or dimethyl sulfoxide (DMSO; used as solvent). Using a modified biotin switch method, SNO residues of mitochondrial proteins were labeled with biotin (**a**). Following mitochondrial isolation by Percoll gradient ultracentrifugation, purity of SSM and IFM preparations was determined by the absence of immunoreactivity for antibodies directed against markers of the plasma membrane (Na^+^/K^+^-ATPase), sarcoplasmic reticulum (SERCA2 ATPase), nucleus (HDAC), cytosol (GAPDH), and enrichment of mitochondrial proteins (VDAC, MnSOD, and TOM20) (**b**). After precipitation of biotin labeled SNO-modified proteins, Western blot analysis was performed for mtCx43 (**c**). Biotin labeled mitochondria were treated with 10 mmol DTT (**c**) or 100 mmol DTT (**d**) and used as a negative control. Band signal intensity of purified SNO-modified mtCx43 was normalized to a loading control taken from the starting mitochondrial lysate representing the total amount of loaded mtCx43 (**c**, **d**). Additionally, SNO of mtCx43 was quantified subsequent to injection of sodium nitrite or an equal volume of NaCl into mouse left ventricles (**f**, **g**). *Asterisk* (*p* < 0.05) indicates significant difference to unmarked groups. Results are expressed as mean ± SEM of seven replicates per group

A modified biotin switch method was utilized for labeling SNO modified proteins with BIAM in mitochondria isolated by Percoll gradient centrifugation (Fig. 6a). Purity of mitochondria was determined by the absence of immunoreactivity for antibodies directed against markers of the plasma membrane (Na^+^/K^+^-ATPase), sarcoplasmic reticulum (SERCA2 ATPase), nucleus (HDAC), and cytosol (GAPDH) (Fig. 6b). Following precipitation and Western blot analysis, intensity of the 43 kDa bands representing SNO-modified mtCx43 was significantly increased in mitochondria incubated with NO donors. In total, samples which were treated with GSNO, SNAP, or a combination of NO donor and Cx43 hemichannel blocker CBX showed an increase (109.2 ± 21.5 %, *n* = 7, *p* < 0.05) in SNO modifications of mtCx43 compared to mitochondria not treated with a NO donor. A hemichannel blocker was also applied to exclude NO-interfering properties of CBX. The specificity of the biotin switch methods was proven by adding DTT to NO donor treated and already biotin-labeled samples leading to a breakdown of disulfide bounds. In samples treated with DTT and a NO donor (SNAP), the amount of SNO modified mtCx43 was significantly reduced by 59.7 ± 7.6 % compared to NO treated samples and reduced by 15.8 ± 7.8 % (*n* = 7, *p* < 0.05) compared to untreated samples (Fig. 6c, e). Application of 100 mmol DTT removed nearly completely the biotin label at Cx43 cysteine residues (Fig. 6d).

Also pharmacological preconditioning by injection of 48 nmol nitrite into the mouse left ventricular cavity in vivo led to an increased SNO modified mtCx43 level by 59.3 ± 18.2 % (*n* = 6, *p* < 0.05) compared to control injected mouse hearts (Fig. 6f, g).

**Fig. 7:**
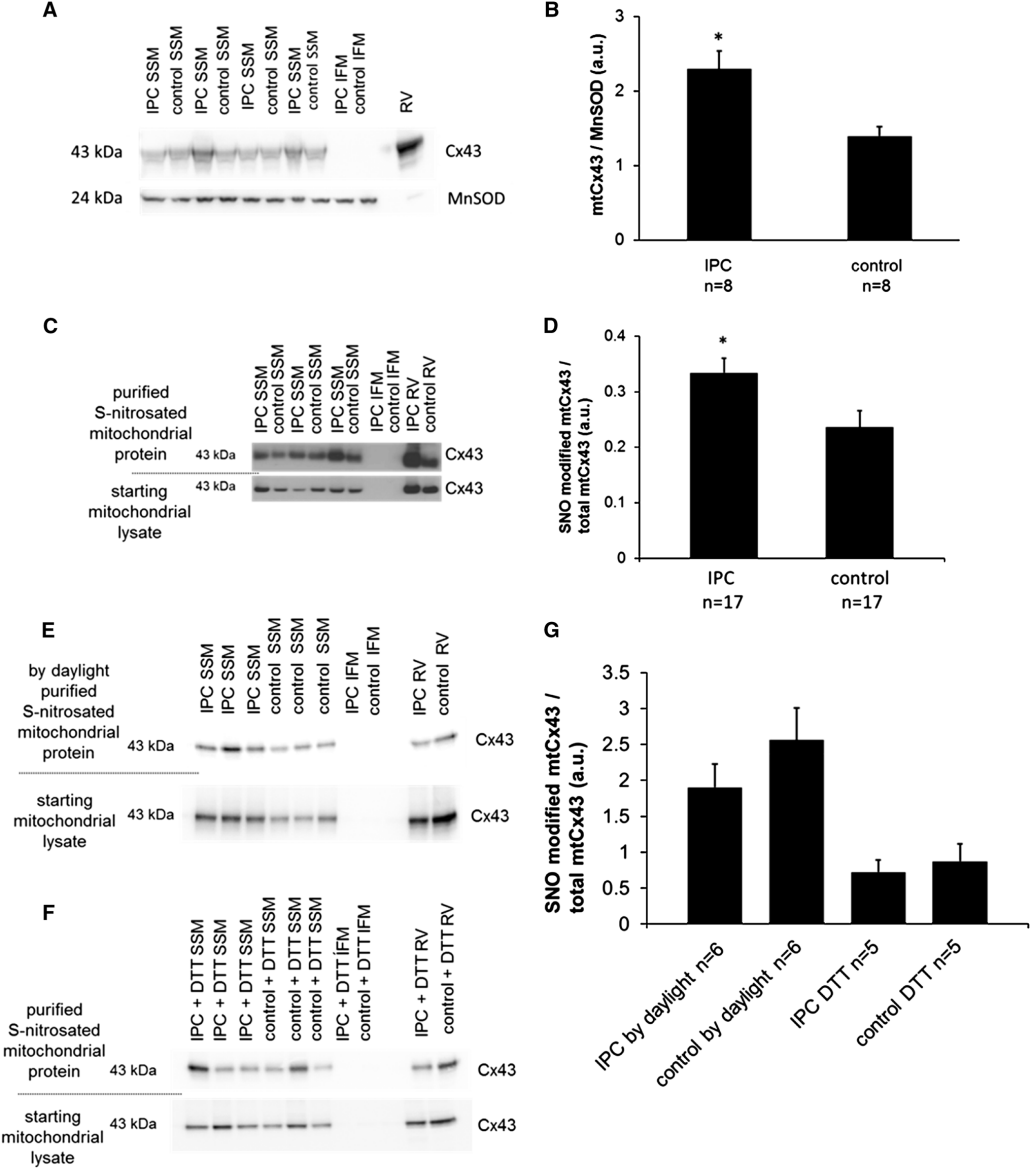
SNO quantification of mtCx43 after IPC. Rat hearts were assigned to a Langendorff perfusion model. One group was preconditioned the other control perfused. SNO quantification of mtCx43 was performed on mitochondria isolated by Percoll gradient ultracentrifugation. Western blot analysis showed that Cx43 is increased following IPC by normalizing mtCx43 to the mitochondrial protein MnSOD (**a**, **b**). Using a modified biotin switch method, SNO residues of mitochondrial proteins were labeled with biotin. Following precipitation of biotin labeled SNO-modified proteins, Western blot analysis was performed for mtCx43 (**c**, **e**, **f**). SNO quantification was performed on mitochondria from IPC and control perfused hearts (**c**, **d**). IFM mitochondria were used as a negative control. Rat RVs were used as positive controls for SNO quantification of mtCx43 from IPC and control hearts. Band signal intensity of purified SNO-modified mtCx43 was normalized to a loading control taken from the starting mitochondrial lysate representing the total amount of loaded mtCx43. In addition, as negative controls, Langendorff experiments were repeated by daylight removing light sensitive SNO modification (**e**) and isolated mitochondria were treated with DTT removing SNO-biotin labels showing the specificity of the biotin switch method (**f**). *Asterisk* (*p* < 0.05) indicates significant difference between groups. Results are expressed as mean ± SEM of 14 replicates of IPC and control perfused hearts (**d**). Five to six replicates per group of negative controls were performed (**g**)

To investigate if SNO increases also with IPC, rat hearts were assigned to a Langendorff perfusion protocol. One group of hearts was preconditioned, while the other group was control perfused and the amount of SNO-modified mtCx43 was quantified for each group by using the modified biotin switch method on pure mitochondria for labeling SNO-modified cysteines. First, Western blot quantification of mtCx43 per mitochondrion confirmed previously published data showing that the mitochondrial amount of Cx43 is increased by IPC (Fig. 7a, b). Second, the percentage of SNO-modified mtCx43 was increased by 41.6 ± 1.7 % (*n* = 17, *p* < 0.05) in preconditioned rat hearts compared to control perfused hearts (Fig. 7c, d). The increase in light sensitive SNO modification was lost by performing the Langendorff perfusion by daylight (*n* = 6, *p* = ns) or by adding 10 mmol DTT after SNO labeling (*n* = 5, *p* = ns) (Fig. 7e–g).

### NO-induced changes mtCx43 phosphorylation

**Fig. 8:**
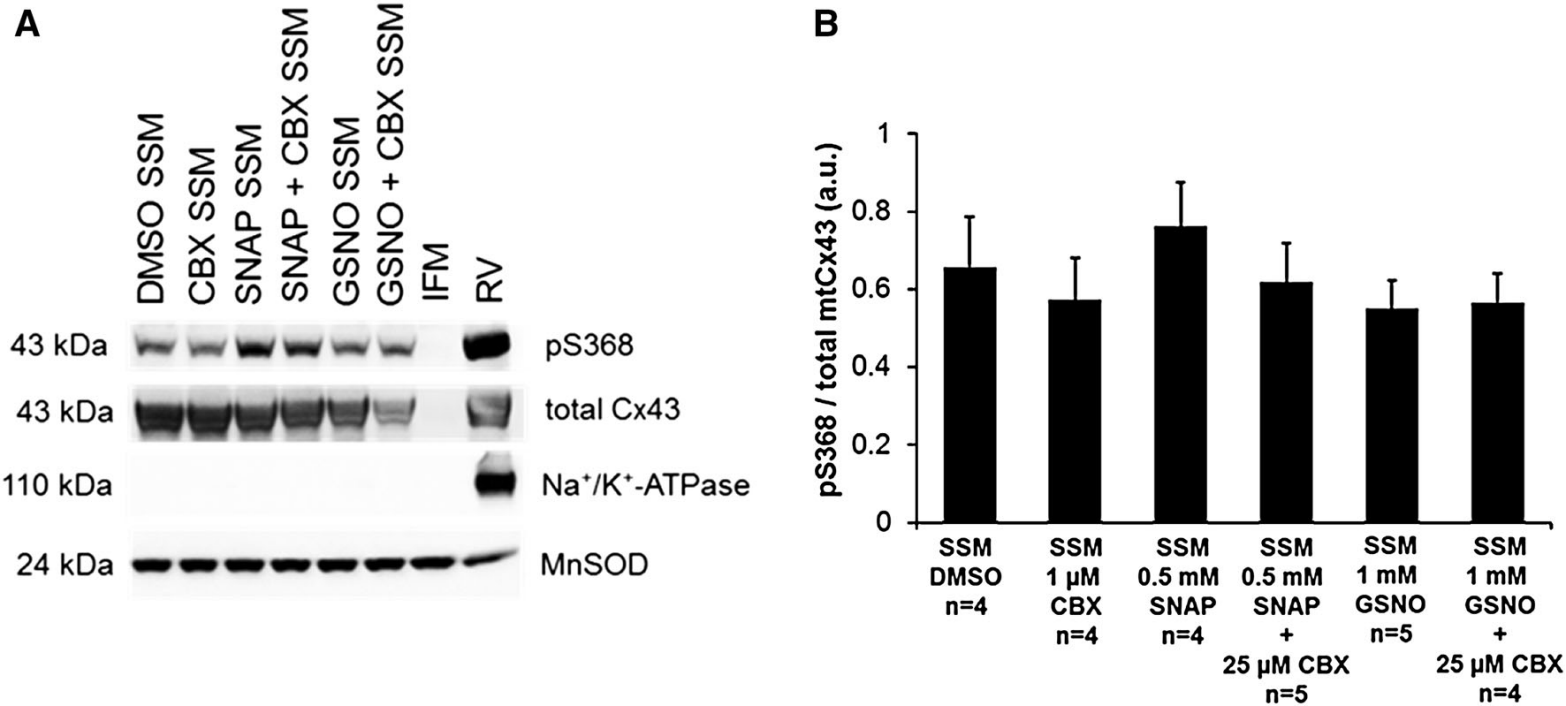
Analysis of Cx43 (S368) phosphorylation. Quantification was performed on mitochondria treated either with carbenoxolone (CBX), *S*-nitroso-*N*-acetyl-dl-penicillamine (SNAP), *S*-nitrosoglutathione (GSNO), a combination of NO donor and hemichannel blocker (SNAP + CBX; GSNO + CBX), or dimethyl sulfoxide (DMSO; used as solvent). Following mitochondrial isolation by Percoll gradient ultracentrifugation, purity of SSM and IFM preparations was determined by the absence of immunoreactivity for antibodies directed against markers of the plasma membrane (Na^+^/K^+^-ATPase) and increase of mitochondrial protein (MnSOD). Western blots were probed for Cx43 phosphorylated at S368 (pS368) as well as for total mtCx43 (**a**). Signals were quantified and expressed as a ratio of phosphorylated serine to total mtCx43 (**b**). *Asterisk* (*p* < 0.05) indicates a significant difference in comparison with unmarked groups. Results are expressed as mean ± SEM of 4–5 replicates per group

Changes in the extent of mtCx43 phosphorylation were analyzed after exposure to NO donors and the hemichannel blocker CBX. SSM were exposed either to 1 µmol CBX, 0.5 mmol SNAP, 1 mmol GSNO, a combination of a NO donor and CBX, or 5 µl DMSO used as a vehicle. Western blot analyses identified no changes in mtCx43 phosphorylation for serine residue 368 (S368) in response to the application of the NO donors or CBX (*n* = 4, *p* < 0.05) (Fig. 8).

## Discussion

Mitochondrial LY uptake and the velocity of K^+^ refilling are increased after the application of the NO donors SNAP and GSNO in SSM, but not in IFM. These increases are abolished by the Cx43 hemichannel blocker, CBX. In addition, ROS production is increased following application of NO donors in SSM, but not in IFM. The ROS increasing effect of NO donors was abolished by hemichannel uncoupler CBX and due to blocking hemichannel opening with Cx43 mimetic peptide Gap26. Furthermore, SNO modification of mtCx43 is increased not only by NO donors, but also by IPC. These new findings not only indicate that SNO regulates Cx43 dependent K^+^ flux which explains the increase in ROS formation [39], but also demonstrate that the SNO increase of mtCx43 following ischemic preconditioning (IPC) may have a role in the signal transduction cascade of cardioprotection. This is supported by results showing an increase in mitochondrial permeability of LY dye following IPC.

A number of studies have shown that Cx43 forms hemichannels at the plasma membrane, which is important for volume regulation of cells and the release of metabolites [19, 24, 42, 45, 54]. Hemichannels are predominantly closed during resting conditions. Changes in the phosphorylation status of the protein and changes in the redox state can increase the probability of channel opening [50, 56]. Cx43 is also present at the inner mitochondrial membrane with N- and C-termini located in the intermembrane space [5]. Cross-linking studies suggest the presence of Cx43 hexamer-like structures in mitochondrial membranes. These findings were supported by reduced mitochondrial LY uptake in the presence of hemichannel blockers carbenoxolone and heptanol and reduced mitochondrial K^+^ influx in the presence of the hemichannel blocker 18α-glycyrrhetinic acid [36]. The Cx32 isoform has reduced K^+^ permeability compared to Cx43. Experiments using a knock-in mouse model, in which Cx43 is absent and replaced by Cx32, also show a reduction in mitochondrial K^+^ and LY permeability, which was not influenced by hemichannel blockers [43]. Our present study confirmed these previous findings of reduced mitochondrial LY uptake in the presence of the hemichannel blocker carbenoxolone, again suggesting the presence of Cx43 hemichannels within the inner mitochondrial membrane of SSM.

The open probability of Cx43-formed GJs of myoendothelial cells is increased by SNO of cysteine 271 and denitrosation by compartmentalized *S*-nitrosoglutathione reductase decreases of GJ permeability [66]. Local NOS can generate NO in specific cellular regions, leading to organelle-specific SNO of proteins that can regulate intracellular processes [26, 44]. In cardiomyocytes, specific NOS isoforms are present in distinct intracellular localizations allowing NO to have a coordinated and restricted interaction with colocalized effectors [21, 82].

In mice hearts, eNOS is involved in the cardioprotection afforded by ischemic preconditioning [74]. Indeed, both NOS inhibitors and genetic deletion of eNOS abolish ischemic preconditioning’s cardioprotection. ENOS is localized in caveolae and transported from the sarcolemma towards the mitochondria [16], in which direct nitrosation of (mitochondrial) proteins, rather than the activation of the soluble guanylate cyclase pathways, appears to be important [48, 67, 68]. There is also evidence that there is not only NO generation by NOS isoforms but also a non-enzymatically release of NO from nitrite [9], which has been suggested to be associated with cardioprotection [64, 65].

In the present study, we used SNAP and GSNO as NO sources. SNAP is the most commonly used nitrosothiol (RSNO), but has an exogenous character, whereas GSNO is an endogenous RSNO. Glutathione (GSH) is a key regulator of the redox state and reduced glutathione is covalently attached to thiols, which is a process called S-glutathionylation [7, 47, 60]. GSNO can lead to both S-nitrosation as well as S-glutathionylation in proteins and it is postulated that a fast transnitrosation reaction occurs before inactivation by glutathionylation [40]. However, the physiological baseline concentrations of GSNO are in the picomolar/nanomolar range in biological fluids like plasma [17, 76]. SNAP and GSNO are very poor NO donors corresponding to NO concentrations of 0.6 and 0.12 % [14]. Therefore, the actual NO concentration applied in this study is much lower than the given NO donor concentrations and may be in the nmol range. Indeed 48 nmol sodium nitrite injection into the LV of a mouse heart and IPC led to an increased SNO of mtCx43 supporting the importance of the observed alterations. Application of 48 nmol, 1 µmol, and 1 mmol GSNO resulted in an increase of SNO modified mtCx43 (data not shown), with no major difference between the low and the high GSNO concentration. Thus, even in the nmol concentration an exogenous NO donor appears to have a greater impact on SNO of mtCx43 as compared to IPC. However, IPC activates several endogenous signaling pathways leading to cardioprotection, one of which is SNO of mitochondrial proteins including mtCx43.

The results obtained by dye uptake were confirmed by measurement of K^+^ influx, which was increased by NO donors and blocked by CBX. During these measurements, MPTP opening was blocked by adding cyclosporine A [22], the proton channel of the ATP-synthase was blocked with oligomycin, and opening of ATP-dependent potassium (mitoK_ATP_) channels were blocked by glibenclamide [62, 72]. However, even in the presence of these inhibitors and CBX a mitochondrial K^+^ influx was still detected. Several other channels other than mitoK_ATP_ channels can contribute to mitochondrial K^+^ fluxes such as calcium-dependent potassium channels, mitochondrial Kv1.3 potassium channels, and the two-pore potassium channels TASK-3 [73]. These data suggest that in the presence of NO, an increase in mitochondrial K^+^ permeability occurs, likely due to increased Cx43 hemichannel opening.

The relevance of SNO-mediated mitochondrial hemichannel opening under more physiological conditions was proven by repeating the experiments without additional blockers, which showed a Cx43-mediated K^+^ influx of significant relevance. Surprisingly, the NO donors did not have an effect on Na^+^ fluxes. It has often been suggested that gap junctions are non-ion specific channels and Cx43 is described as discriminating by size and has a negligible charge-dependent selectivity [29]. It is also reported that in cardiac mitochondria, the Na^+^ influx is mainly achieved by the Na^+^/Ca^2+^ exchanger [30] and that Na^+^ is exported by mitochondrial Na^+^/H^+^ exchange [28]. Metabolic inhibition leads to an increase in matrix Na^+^, whereas in energized mitochondria the matrix Na^+^ is lower than cytosolic Na^+^ [13, 28]. Therefore, the state 4 respiration of mitochondria might have contributed to the lack of sodium influx in the present study.

The data indicate that mitochondrial permeability is increased in the presence of the NO donors SNAP and GSNO, especially for K^+^. This increase is abolished by the hemichannel blocker CBX. Western blot analysis confirmed an increase in SNO of mtCx43 following the application of both NO donors, which indicates that SNO of mtCx43 could lead to opening of Cx43 hemichannels at the inner mitochondrial membrane.

Opening of hemichannels at the sarcolemma contributes to myocardial damage following ischemia/reperfusion [79] and dephosphorylation of S368 increases the permeability of hemichannels at the plasma membrane. S368 is phosphorylated by protein kinase C which is localized in mitochondria [32, 33, 41]. It is possible that mtCx43 hemichannel opening is induced or supported via a SNO-mediated decrease of mitochondrial protein kinase activity. In this study, Western blot analysis of mtCx43 phosphorylation, however, showed no significant changes in the phosphorylation of S368 induced by the NO donor SNAP or GSNO. It must, however, be acknowledged that only one out of many (up to 21) epitopes being phosphorylated was analyzed in the present study.

SNO of mtCx43 was also increased in mitochondria treated with NO donors (SNAP and GSNO) and the hemichannel blocker CBX. CBX is a glycyrrhetinic acid derivative, which at low concentrations (1–25 µM) is a gap junction and hemichannel uncoupling agent. Accordingly, CBX blocks the ion fluxes and their downstream effects mediated by mtCx43 hemichannels, but not SNO of mtCx43 per se [10, 11, 51, 52].

IPC, a mechanism protecting the heart from ischemia/reperfusion injury, leads to inhibition of MPTP opening and prevents cell death [24, 38]. In Cx43-deficient (Cx43^+/−^) mice hearts [61] and in cardiomyocytes isolated from Cx43-deficient hearts, IPC-induced cardioprotection was abolished [34]. Inhibition of the import of Cx43 to mitochondria attenuated the diazoxide-induced cardioprotection, confirming the important role of mtCx43 in cardioprotection [53]. MtCx43 is proposed to contribute to increased K^+^ influx into the mitochondrial matrix [6] and cardiomyocyte ROS formation [23], thereby mediating cardioprotection independent of reperfusion injury salvage kinase (RISK) and survivor activating factor enhancement (SAFE) signaling [57].

In the present study, ROS formation was increased following application of low and high doses of NO donors for ADP stimulated complex 1 respiration of SSM. This effect was blocked by CBX or by Cx43 mimetic peptide Gap26, which indicates that subsarcolemmal mitochondrial ROS formation is induced by NO activation through mtCx43. ROS triggering cardioprotection must be viewed separately from ROS contributing to irreversible injury following sustained episodes of ischemia/reperfusion. Chouchani et al. [8] showed that SNO of a cysteine residue on mitochondrial complex 1 has a cardioprotective effect by reducing ROS formation. In the present study, ROS formation of energized and non-energized IFM was also decreased by application of NO donors and not altered by CBX. These data demonstrate that the second messenger NO can have opposite effects in the two different populations of mitochondria.

The application of FCCP, which permeabilizes the inner mitochondrial membrane to protons thereby uncoupling the electron transport system from oxidative phosphorylation [25], decreased ROS formation [37, 75, 80] to a similar extent in all treatment groups. These experiments suggest that the increase in ROS formation by NO is induced by mitochondrial respiratory chain complexes using the proton gradient across the inner mitochondrial membrane.

In addition, the respiratory chain complex 1 inhibitor rotenone increased mitochondrial ROS production as previously reported [20, 77]. The ROS increasing effect of NO was abolished by rotenone indicating that the regulation of ROS formation by Cx43 could be through interaction with complex 1. Modulation of complex 1, rather than other complexes, by Cx43 has been previously reported for oxygen consumption as well [2].

The present study demonstrates that SNO of mtCx43 influences mitochondrial permeability, K^+^ fluxes, and ROS formation. It is known that increased K^+^ fluxes via opening of mitochondrial K_ATP_ channels and increased ROS formation are relevant for inhibition of MPTP opening during ischemia/reperfusion injury and are thereby two factors necessary for cardioprotection [6, 23, 34, 53, 61]. In addition, this study shows that IPC increases SNO of mtCx43 and mitochondrial permeability, indicating a potential link between NO and mtCx43 in the signal transduction cascade of cardioprotection. Furthermore, pharmacological preconditioning by injection of a low dose sodium nitrite (48 nmol) into the LV of mouse hearts was associated with augmented SNO of mtCx43. However, it remains unclear whether or not SNO of mtCx43 is sufficient for cardiomyocyte preconditioning.

In addition, the sites of mtCx43 that are S-nitrosated and are responsible for mediating increased mitochondrial ion fluxes and ROS formation remain to be identified using cysteine epitope replacement studies. Additional studies are needed to investigate the influence of mitochondrial Cx43 hemichannel-mediated ion fluxes in the context of preconditioning.

## Electronic supplementary material

Supplemental Fig. 1 LY dye uptake of ultrasound treated mitochondria. SSM were either incubated with carbenoxolone (CBX), *S*-nitroso-*N*-acetyl-dl-penicillamine (SNAP), *S*-nitrosoglutathione (GSNO), a combination of a NO donor and hemichannel blocker (SNAP + CBX; GSNO + CBX), or dimethyl sulfoxide (DMSO; used as solvent). After incubation with LY and RITC-dextran, mitochondria were ruptured with ultrasound. Possible dye interaction with membrane fragments were analyzed by measuring LY dye (left panel) and RITC-dextran dye (right panel), which were expressed as arbitrary units of fluorescence. Data are shown as mean ± SEM of 7 replicates per group of ultrasound treated mitochondria from rat left ventricles.

Supplemental Fig. 2 Mitochondrial Na + permeability. Analysis of Na + influx was measured in SSM from the left ventricles of rat hearts. Mitochondria were either treated with carbenoxolone (CBX), *S*-nitroso-*N*-acetyl-DL-penicillamine (SNAP), *S*-nitrosoglutathione (GSNO) a combination of a NO donor and hemichannel blocker (SNAP + CBX; GSNO + CBX), or dimethyl sulfoxide (DMSO; used as solvent). Oligomycin, glibenclamide, and cyclosporine were present during the entire experiment to measure Na + influx exclusively via Cx43 hemichannels. The rate of SBFI fluorescence ratio (340/380 nm) change from mitochondria in different treatments was estimated for the initial 2 s after the addition of 10 mmol (A) and 140 mmol (B) NaCl pulses. Data correspond to mean ± SEM of 5 replicates per group.

Below is the link to the electronic supplementary material.

Supplementary material 1 (PPTX 276 kb)
