# Supplementary figures and images for: S-nitrosation of mitochondrial connexin 43 regulates mitochondrial function
Source: Basic Res Cardiol. 2014 Aug 13;109(5):433. doi: 10.1007/s00395-014-0433-x (PMC4168224; doi:10.1007/s00395-014-0433-x)

## Slide 1
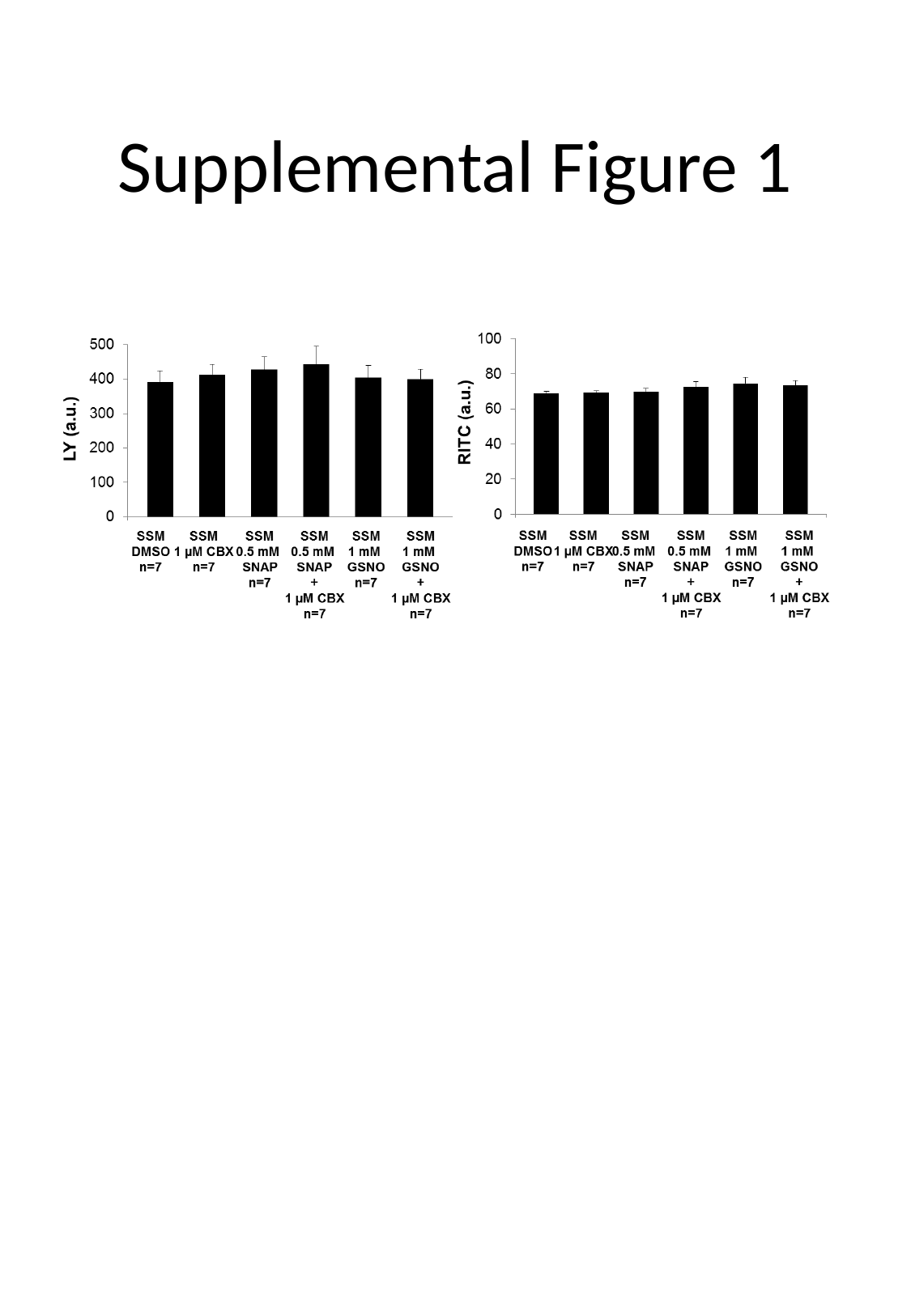

# Supplemental Figure 1

## Slide 2
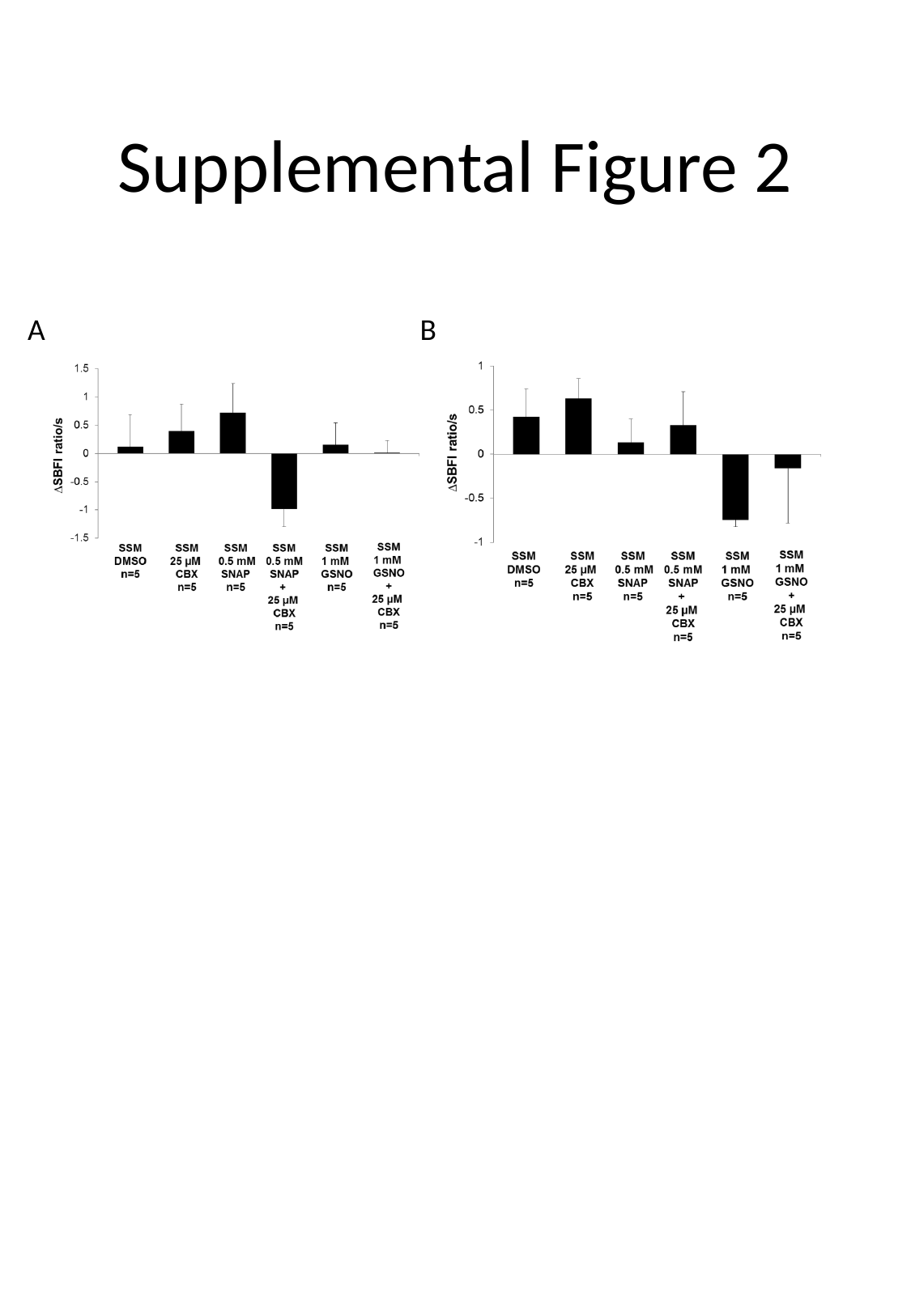

# Supplemental Figure 2
A
B

Supplement: Supplementary file 1 — Supplementary material 1 (PPTX 276 kb) [file 395_2014_433_MOESM1_ESM.pptx]
